# Hypoplastic Left Heart Syndrome: A New Paradigm for an Old Disease?

**DOI:** 10.3390/jcdd6010010

**Published:** 2019-02-23

**Authors:** Paul Grossfeld, Shuyi Nie, Lizhu Lin, Lu Wang, Robert H. Anderson

**Affiliations:** 1 Division of Cardiology, Department of Pediatrics, UCSD School of Medicine, La Jolla, CA 92093, USA; lilin@ucsd.edu (L.L.); luw059@ucsd.edu (L.W.); 2 School of Biology, Georgia Institute of Technology, Atlanta, GA 30332, USA; shuyi.nie@biology.gatech.edu; 3 Cardiovascular Research Centre, Institute of Genetic Medicine, Newcastle University, Newcastle upon Tyne NE1 7RU, UK; sejjran@ucl.ac.uk

**Keywords:** hypoplastic left heart syndrome, Jacobsen syndrome, neural crest cell, endocardium, cardiac myocyte, hyperplasia

## Abstract

Hypoplastic left heart syndrome occurs in up to 3% of all infants born with congenital heart disease and is a leading cause of death in this population. Although there is strong evidence for a genetic component, a specific genetic cause is only known in a small subset of patients, consistent with a multifactorial etiology for the syndrome. There is controversy surrounding the mechanisms underlying the syndrome, which is likely due, in part, to the phenotypic variability of the disease. The most commonly held view is that the “decreased” growth of the left ventricle is due to a decreased flow during a critical period of ventricular development. Research has also been hindered by what has been, up until now, a lack of genetically engineered animal models that faithfully reproduce the human disease. There is a growing body of evidence, nonetheless, indicating that the hypoplasia of the left ventricle is due to a primary defect in ventricular development. In this review, we discuss the evidence demonstrating that, at least for a subset of cases, the chamber hypoplasia is the consequence of hyperplasia of the contained cardiomyocytes. In this regard, hypoplastic left heart syndrome could be viewed as a neonatal form of cardiomyopathy. We also discuss the role of the endocardium in the development of the ventricular hypoplasia, which may provide a mechanistic basis for how impaired flow to the developing ventricle leads to the anatomical changes seen in the syndrome.

## 1. Introduction

Hypoplastic left heart syndrome, if untreated surgically, is a uniformly fatal lesion. It occurs in about 3% of all infants born with congenital cardiac disease. The first report describing such a patient was provided in 1851 by Dr. Bardeleben, a German pathologist [1]. It was Lev who was first to describe the constellation of lesions making up the syndrome, although he described them in terms of hypoplasia of the aortic tract [2]. Noonan and Nadas then grouped together the anatomic findings to define a specific syndrome [3]. Recently, a 6500-year-old infant mummy discovered in Peru was found to have a comparable cardiac anatomy [4]. In the United States, between 1000 and 2000 infants are born annually with the syndrome.

## 2. Phenotypic Features

In anatomic terms, the syndrome represents an acquired disease of fetal life, since by definition, the ventricular septum is intact [5]. Recognition of the precise phenotypic features of those with the syndrome is important, since hypoplasia of the left ventricle can be found in several other settings. These include patients with unbalanced atrioventricular canal defect, double outlet right ventricle, discordant ventriculo-arterial connections, or common arterial trunk. In these cases, the ventricular septum is deficient. Hence, these are distinct disease entities not only phenotypically but also mechanistically. There is then further phenotypic variability among the group of patients properly classified as having hypoplastic left heart syndrome. This depends on the variability in the morphology of the mitral and aortic valves, the degree of hypoplasia of the left ventricle, and the size of the aorta. Not surprisingly, this degree of phenotypic variability has hindered studies aimed at understanding the molecular mechanisms underlying the syndrome and may also influence the clinical management. In addition to the left-sided problems in those with the syndrome, the atrial chambers and the extra-pericardial aortic pathways are frequently abnormal.

**Figure 1:**
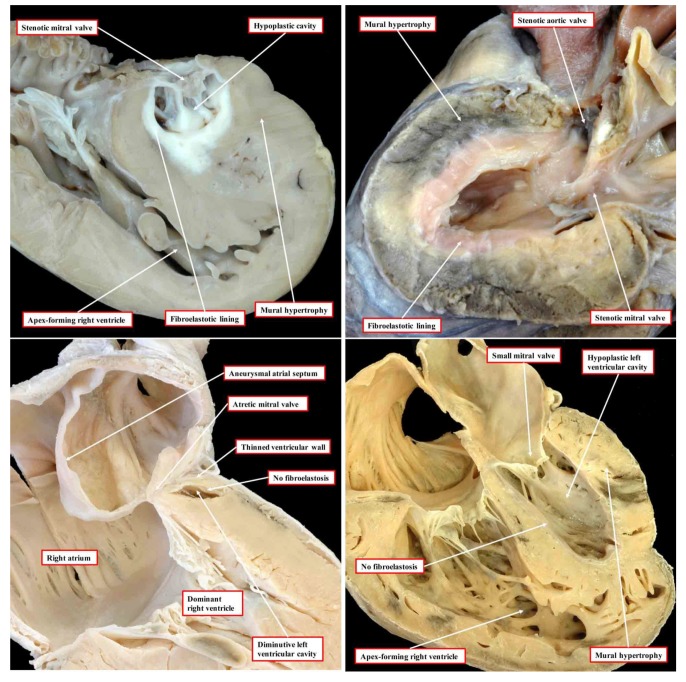
The images, all photographed by Diane E. Spicer and reproduced with her permission, show the phenotypic variants of hypoplastic left heart syndrome as seen in the clinical setting. The upper left panel shows the variant with mitral stenosis and aortic atresia. The heart in the upper right-hand panel has mitral and aortic stenosis. In the lower panels, to the left is seen the variant with mitral atresia, and to the right is the rarest variant with left ventricular hypoplasia with the small aortic and mitral valves, their size in keeping with that of the left ventricle although the aortic valve is not seen in the four-chamber section through the heart.

In the commonest variant of the syndrome, the left ventricular wall is severely thickened, resulting in diminution of the size of the left ventricular cavity, which cannot sustain systemic cardiac output (Figure 1, upper left-hand panel).

This variant is associated with stenosis of the mitral valve and usually with aortic atresia. The typical feature of the hypoplastic left ventricle in this variant is the thick layer of fibroelastosis that lines its cavity. In the setting of aortic atresia, the hypoplastic ascending aorta serves as no more than a conduit, which feeds the coronary arteries in retrograde fashion. In some instances, nonetheless, the aortic valve can be critically stenotic rather than atretic but still with a thick walled and hypoplastic left ventricle, which again has a fibroelastotic lining (Figure 1, upper right-hand panel). In the second commonest phenotypic pattern, the mitral valve is atretic rather than stenotic (Figure 1, lower left-hand panel). This variant is always associated with aortic atresia. Because of the mitral atresia, blood has never been able to enter the left ventricle. In consequence, the cavity of the ventricle is slit-like or exceedingly diminutive. Its walls are much thinner than is the case when the mitral valve is stenotic. The endocardial lining of the left ventricle also lacks the fibroelastotic layer characteristic of mitral stenosis (see upper panels of Figure 1). There is then a third variant, which is the least frequent. In this type, the mitral and aortic valves are both small, but their size is in keeping with that of the hypoplastic left ventricle (Figure 1, lower right-hand panel). This variant has been described as the “hypoplastic left heart complex” [6]. Some would describe the entity as no more than a severe aortic coarctation with associated left ventricular hypoplasia, since it is the obstructed aortic arch that is the most obvious feature of the pattern. The majority of the hearts initially described by Noonan and Lev were of this type. Examples of this variant, nonetheless, are to be found in historical archives having been classified as representing “hypoplastic left heart syndrome” by the pathologists making the initial diagnosis [5]. Of greater significance is that, to date, the mechanisms underlying the development of any of the different variants remain unknown.

## 3. Clinical Progression

Without intervention, the syndrome is universally fatal, usually within the first weeks of life. Surgical treatment is palliative, consisting of three surgical interventions in the first few years of life. The first, the Norwood procedure, is performed in the neonatal period, followed by the construction of a Glenn anastomosis at between 4 and 6 months of age, with conversion to the Fontan circulation achieved at around the age of 3 years. These interventions serve to convert the heart to function as a two-chambered entity, with the morphologically right ventricle serving as the systemic ventricle. A small subset of infants may be treated by cardiac transplantation. This option, however, is not viable for most infants born with the malformations because of the limited number of donor hearts. Overall, the syndrome is the greatest single cause of morbidity and mortality in infants born with congenital cardiac disease. 

The long-term prognosis is guarded. This is because, over time, the systemic right ventricle may fail, leading to premature death without additional interventions. The only viable option then for long-term survival is heart transplantation. The timing for the listing of patients for transplantation, however, remains controversial and, often times, is unclear. Not uncommonly, patients suffering right heart failure will die before receiving a donor heart. The syndrome and its subsequent treatment, therefore, place an enormous burden on families. It can result in significant loss of time from work for the parents, coupled with extraordinary psychosocial stressors. All things considered, the syndrome is the single most expensive congenital cardiac entity, with an estimated direct annual cost exceeding $1 billion in the United States of America for this disease [7].

## 4. Existing Concepts for Abnormal Morphogenesis 

Two contrasting theories have been proposed to explain the pathogenesis. The first, which implicates abnormal flow, suggests that the reduced size of the left ventricular cavity is secondary to decreased flow across the mitral valve, with decreased growth of the distal left-sided structures. In support of this theory, impaired ventricular growth has been shown in animal models in which there has been compromise of the anterograde flow to the developing ventricle. These models have revealed decreased proliferation of the left ventricular cardiomyocytes [8]. Relief of an obstructed aortic valve during fetal life in humans, however, does not, in most cases, prevent the ongoing development of the syndrome [9]. In the majority of fetuses developing the features of the syndrome after this procedure, endocardial fibroelastosis was present in the setting of mitral stenosis. This is a marker for endocardial injury, suggesting that additional factors contribute to the left ventricular hypoplasia. It is also possible, nonetheless, that any ability to respond to the restoration of normal hemodynamics has been lost by the time of intervention. For example, primary cilia that are required for sensing fluid shear stress are resorbed on murine endocardial cells at E12.5. Mutations in several genes involved with ciliary function, such as *Kif3a, Lrd, Pkd2,* and *Ift88*, result in defective maturation of the developing left ventricle [10]. As yet, it is unknown at what developmental stage this process occurs in the human fetus.

The second theory implicates the hypoplasia of the left ventricle as the primary mechanism. In this regard, it is known that some patients have significant left ventricular hypoplasia in the absence of significant mitral or aortic stenosis. Consistent with this hypothesis, mutations in at least one gene, namely *NKX2-5*, have been reported in association with both hypoplasia of the left heart and cardiomyopathy [11,12]. There are also reports of patients with the syndrome exhibiting so-called “non-compaction” of the right ventricle. In reality, this process reflects excessive right ventricular trabeculation. Such observations provide further evidence to suggest that the syndrome may be a neonatal primary cardiomyopathy [13].

At the cellular level, increased fibrosis associated with disarray of the cardiomyocytes has been observed in tissue obtained from explanted hearts from infants [14]. The specific anatomic combinations in the studied specimens, however, were not stated [14]. More recently, left ventricular tissue was obtained from hearts of fetuses with the syndrome, aged from 20 to 27 weeks [15]. Those studying this material reported decreased cardiac progenitors, with reduced number of cardiomyocytes and endocardial cells. They also observed increased numbers of smooth muscle cells and myofibroblasts. Molecular analysis of the tissues demonstrated impaired vasculogenesis, with the upregulation of hypoxia-inducible factor 1 alpha, oncogene-associated cellular senescence, and TGF-beta-associated fibrosis. These studies are particularly significant since, as we have described, at least in the setting of mitral stenosis, left ventricular fibrosis has been found to be an integral component of the syndrome [5]. The critical question remains as to whether the findings are secondary, in other words due to ischemia from altered hemodynamics, or are part of the primary disease process which underscores the ventricular hypoplasia. The answer to the question will surely be provided if it proves possible to unravel the primary cellular and molecular mechanisms underlying the development of left ventricular cavitary hypoplasia and mural thickening during the early stages of cardiac development. This will best be achieved using genetically engineered animal models, providing that the models accurately reflect the phenotypic variability found in the clinical setting (Figure 1). 

## 5. Genetically Engineered Animal Models for Human Genes Implicated in the Syndrome

Although there is compelling evidence implicating a genetic etiology for the syndrome, to date, only a small number of potential disease-causing genes in humans have been identified. As far as we are aware, the genes identified thus far are *NKX2-5* [11], *NOTCH1* [16], *ETS1* [17], *HAND1* [18], and *rbFOX2* [19]. Mutations in these five genes, however, probably account, at most, for a small subset, perhaps one-tenth, of all patients born with the syndrome [20]. In most cases, therefore, the specific genetic etiology remains unknown. This may reflect a multifactorial etiology for the syndrome. In support of this, the Baltimore-Washington Infant Study group has identified environmental factors, such as parental exposure to organic solvents, which are associated with an increased frequency of the syndrome [21]. More recently, epigenetic factors have been implicated, such as those specific for NKX2-5, which suggest that the syndrome may be the consequence of a combination of genetic, epigenetic, and environmental factors [22]. This may also explain the difficulty in generating appropriate genetically engineered animal models for the syndrome. Such animal models, providing they accurately demonstrated the appropriate phenotypic anatomy, would provide an opportunity to gain unprecedented novel insights into some of the earliest molecular and cellular events that occur in the development changes leading to the syndrome. As yet, however, no models have been produced that show left ventricular hypoplasia in the setting of an intact ventricular septum and with concordant atrioventricular and ventriculo-arterial connections.

**Figure 2:**
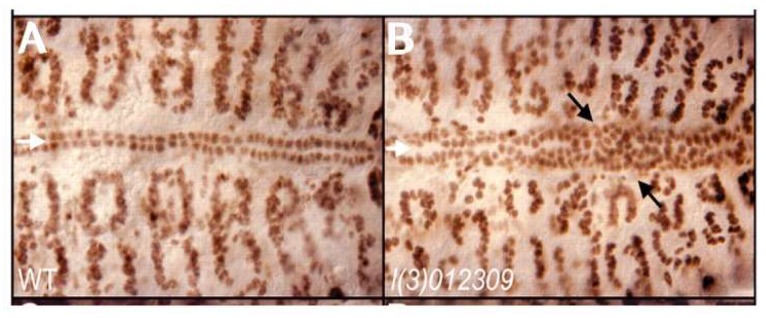
Wildtype (**A**) and ETS-1 Drosophila loss-of-function mutant (**B**) demonstrating increased cardiac myocytes causing the obliteration of the chamber (Reproduced with permission from Alvarez AD, et al., *Development*. 2003;130:3015-26).

Previous studies, nonetheless, have implicated the *ETS1* gene as one cause of left ventricular hypoplasia in humans with Jacobsen syndrome [17]. In this syndrome, one-twentieth of all infants are born with the recognized phenotypic features of hypoplastic left heart syndrome. A de novo *ETS1* loss-of-function mutation has also been identified in one patient with a hypoplastic left ventricle variant and several other clinical features of Jacobsen syndrome [17]. Our own investigations have shown that *ETS1* is expressed in two cellular lineages during murine heart development, namely the neural crest and the endocardium [23]. This suggests that defects in one, or both, of these populations of cells could be responsible for producing the phenotypic findings of the hypoplastic left heart syndrome. It may be pertinent, therefore, that loss-of-function mutations in *ETS1* in early development of the heart in *Drosophila* cause a loss of pericardial cells along with an increased number of cardiomyocytes [24]. This process, furthermore, selectively involves the posterior aspect of the heart tube, which may be analogous to the left ventricle in a mammalian heart (Figure 2). The molecular basis for this selective effect on a posteriorly located subset of cardiomyocytes, however, is unknown. 

**Figure 3:**
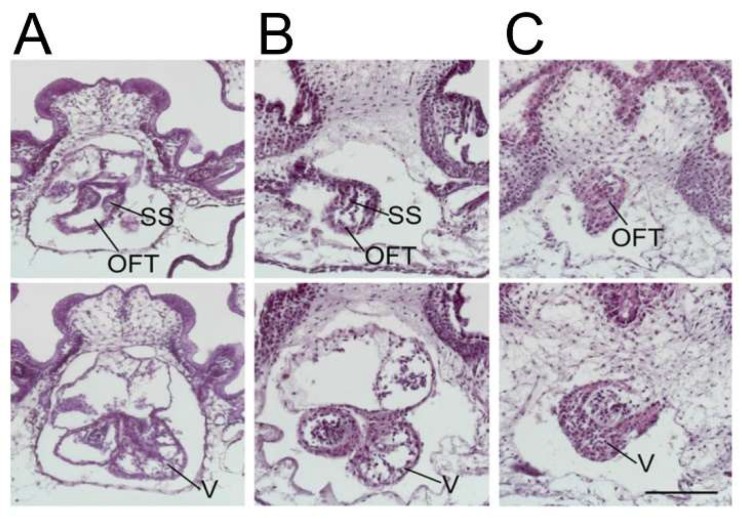
The ETS-1 knockdown (KD) in a frog. (**A**) Wildtype (**B**) neural crest KD, and (**C**) heartfield KD. SS = spiral septum; OFT = outflow tract; and V = ventricle. The knockdown of ETS-1 only in the neural crest results in outflow tract defects, but a normal ventricle indicates that additional factors are required for the development of the “hypoplastic” ventricle. The knockdown of ETS-1 in the cardiac mesoderm causing the “hypoplastic” left ventricle indicates a mesodermally derived factor contributes to the development of the “hypoplastic” left ventricle (Reproduced with permission from Nie S and Bronner S, 2015).

Knockdown of *ETS1* in the cardiac mesoderm in *Xenopus* produced, most commonly, a ventricular phenotype comparable with the morphological findings in the syndrome, specifically an increase in ventricular wall thickness characterized by an expansion of the compact zone and almost a complete loss of the trabecular layer. This was associated with a loss of endocardial cells [25] (Figure 3). 

**Figure 4:**
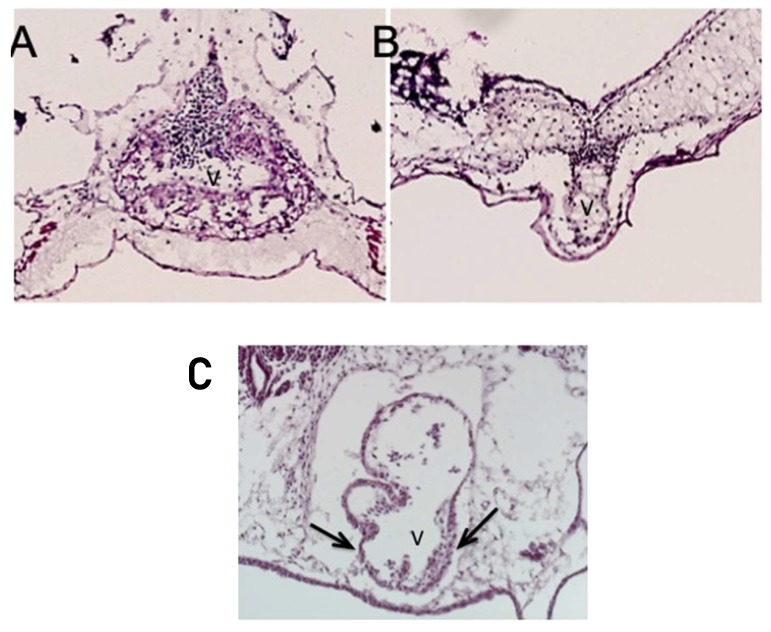
The panels show the result of the knockdown of the cardiac mesoderm-specific ETS-1 knockdown in the frog. The upper left panel (**A**) shows a control heart, while the upper right (**B**) and lower (**C**) show knockdown hearts. The lower panel is particularly significant, with the arrows showing a mixed ventricular phenotype, with differential thinning and thickening of the wall. Abbreviation: V: ventricle.

Less commonly, instead of the thickened ventricular myocardium, the knockdown of *ETS1* in the cardiac mesoderm produced a very immature thin-walled and hypoplastic ventricle, as well as a variant showing an intermediate phenotype. In this latter pattern, the ventricular wall was partially thinned but also thickened (Figure 4, lower panel). 

It is unknown whether the growth-arrested heart is a distinct phenotype or a precursor to the development of the “hypoplastic left heart-like” ventricular phenotype. 

When knocked down in the neural crest, however, a reduction in the levels of *ETS1* caused defects of the aorta and outflow tract but did not affect ventricular development. This parallels the observation in humans, namely that lesions can afflict the aortic valve without having any effect on the ventricular development. Taken together, these results implicate a factor, or factors, originating from the cardiac mesoderm that functions to maintain a balance between the proliferation of the compact and trabecular layers of the ventricular wall by regulating cell proliferation and differentiation and may be required for the endocardium (see below).

**Figure 5:**
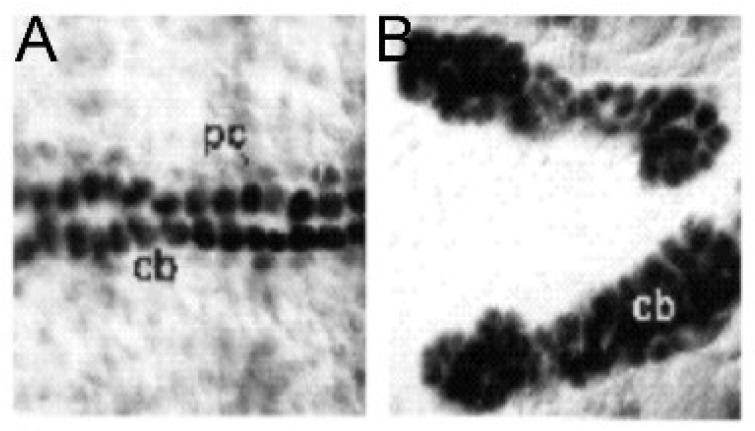
The Drosophila NOTCH mutant heart: (**A**) a normal heart and (**B**) a mutant heart. Similar to ETS1 mutants, there is a loss of pericardial cells (pc) and a gain of cardiac myocytes (cb). (Reproduced with permission from Hartenstein A., et al., 1992).

Mutations in *NOTCH1* have also been reported in association with the syndrome [16]. As is the case with loss-of-function mutations of *ETS1,* mutations in the *NOTCH1* gene were also found to cause an increase in cardiac myocytes in *Drosophila*, along with a complete loss of pericardial cells. The end result is a very abnormal bifid heart with densely packed clusters of cells and a decreased chamber volume [26] (Figure 5). 

**Figure 6:**
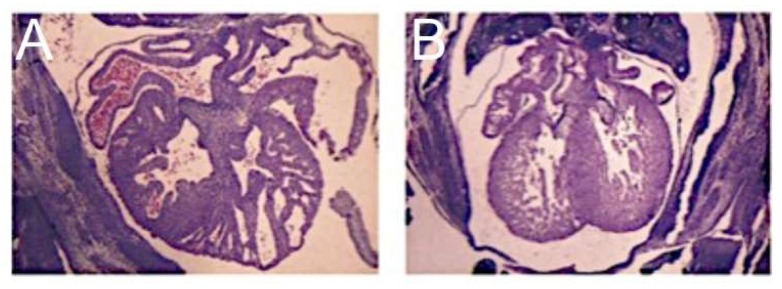
The transgenic overexpression of BMP10 in the developing murine left ventricle (**A**) results in a hypertrabeculated LV in which the chamber volume is severely diminished. The wildtype control is shown in **B**. (Reproduced with permission from Pashmforoush M., et al., 2004).

A mutation in the *NKX2-5* gene has also been reported in association with the syndrome [11]. The conditional deletion of this gene in the murine ventricle produces a cardiomyopathic phenotype with the upregulation of BMP10. The ventricular-specific overexpression of BMP10 in the mouse model, however, produces an increase in the ventricular trabeculations with loss of chamber volume but in the presence of a thinned compact layer [27] (Figure 6). In this model, therefore, the cavitary hypoplasia is due to an overgrowth of the trabecular rather than the compact layer and in the absence of fibroelastosis. These findings bear scant resemblance to the phenotype that includes mitral stenosis as seen in the setting of hypoplastic left heart syndrome (See Figure 1, left panel).

The left ventricular-specific overexpression of BMP10 in the mouse, coupled with the observations following knockdown of ETS1 in *Xenopus*, have parallels with the observations of those that demonstrated a role for Hand2 in regulating the balance between the compact and trabecular components of the murine ventricular walls [25]. In this study, it was shown that the loss of Hand2 in the absence of Hand1 led to a diminished left ventricular cavitary volume due to mural thickening. These investigators also identified ectopic expression of markers in the left ventricular compact myocardium but reduced markers in the trabecular layer [25]. 

## 6. The Endocardium in the Setting of Hypoplastic Left Heart Syndrome

**Figure 7:**
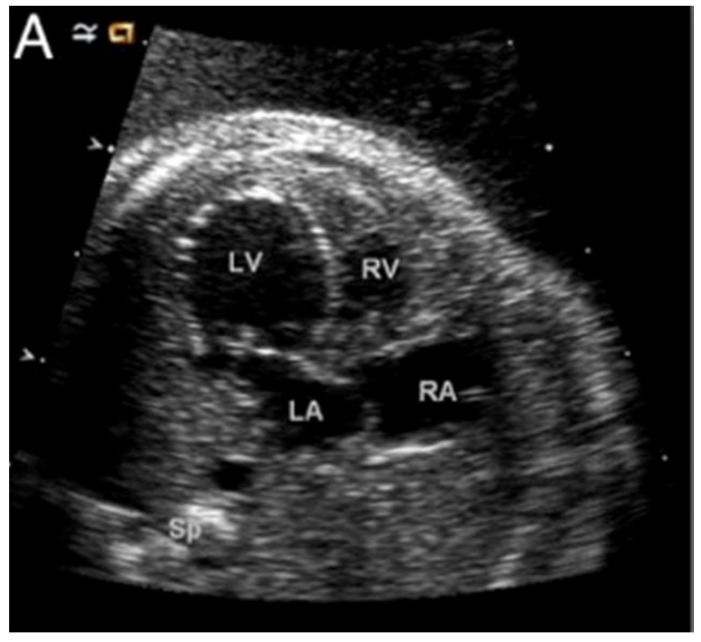
A fetal echo of a 22-week fetus with hypoplastic left heart syndrome here shown with a dilated, apex-forming, poorly functioning left ventricle. Abbreviations: LV: left ventricle; RV: right ventricle; LA: left atrium; and RA: right atrium (Reproduced with permission from Feinstein J., et al., JACC, 2011).

Aortic stenosis is one of the earliest defects to be observed when the hypoplastic left heart syndrome is seen to develop during fetal life [28]. This suggests that, at least in one subset of patients with the syndrome, aortic valvar dysfunction is a prerequisite to the development of the abnormal left ventricle. Figure 7 shows a dilated and poorly functioning left ventricle that is apex-forming at 22 weeks of gestation. With ongoing gestation, this fetus developed the phenotypic findings of the syndrome [28]. 

**Figure 8:**
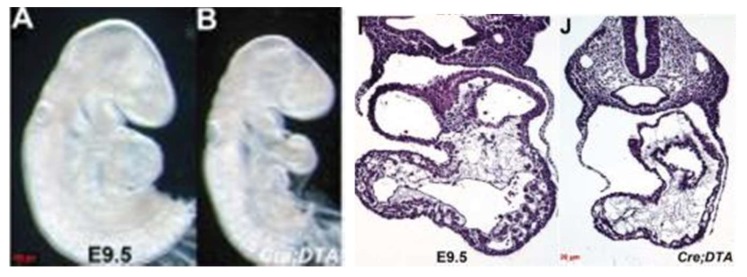
Endocardial-specific genetic ablation in E9.5 mouse embryo. Control: left in each panel; endocadial-deficient embryo: right in each panel (Reproduced with permission from Snider P, et al. J Cardiovasc. Dev. Dis. 2014 Dec;1(3):214-236).

The mechanisms leading to the transition from the dilated and poorly function left ventricle to the typical thick-walled ventricle thus far remain unknown. It is intuitive, nonetheless, to suggest that there is initially an arrest in the growth of the left ventricle compared to the right, followed by a thickening of the left ventricular wall. Relevant to this, a potential direct role for the endocardium in the development of the left ventricle was tested by Conway and his associates [29]. They were able to genetically ablate the endocardium during the murine cardiac development. This was achieved by using an endocardial-specific CRE driver to express diphtheria toxin so as to engineer timed and targeted death of the endocardial cells. The perturbation arrested the growth of the left ventricle, resulting in embryonic lethality by E11 (Figure 8). These embryos appear similar to a subset of ETS1 cardiac mesodermal knockdown embryos shown above in that they exhibit growth arrest in the setting of a thin-walled and small left ventricle.

Loss of endocardial function leading to an initial arrest of growth of the developing left ventricle may be a critical early step in the pathogenesis of the syndrome. This could be the consequence of a primary genetic defect causing the loss of endocardial function. Alternatively, a defect in valvar development could lead to endocardial injury and loss of function, as described above. Studies in rats have provided further support linking impaired hemodynamics and impaired ventricular function to endocardial defects. Thus, distention of the left ventricle caused by surgically induced aortic regurgitation was shown to lead to endocardial injury and endocardial fibroelastosis [30]. We have already cited the studies in the developing chick that show decreased flow across the mitral valve subsequent to ligation of the left atrium can produce a variant of left ventricular hypoplasia [8]. More recently, it was shown that the decreased inflow of blood caused hypoxia [31]. This, in turn, produced endocardial fibroelastosis with subsequent cardiomyocytic hyperplasia. These findings are much more in keeping with the phenotypes observed in the clinical setting (Figure 1). Studies in zebrafish have also demonstrated that, during early development, diminished left ventricular function leads to impaired development of both the atrioventricular and arterial valves [32].

## 7. Neural Crest Cells and Hypoplastic Left Heart Syndrome

The fact that ETS1 is expressed in both the endocardium and neural crest has raised the possibility that one, or both, of these lineages could be involved in producing the syndrome. In humans, there is reported to be a 117-fold higher frequency of Hirschsprung’s disease, a neural crest disorder, in patients with hypoplastic left heart syndrome [33]. This suggests that impaired function of the neural crest could, indeed, underlie this association. Our experiments using ETS1 knockout mice, however, did not produce the anticipated phenotypes of hypoplastic left heart syndrome. Instead, loss of ETS1, with very high penetrance, produced mice with a double outlet right ventricle. This was dependent on the genetic background. It has also been shown, nonetheless, that parents of patients with the syndrome have a significantly increased frequency of isolated dilation of the ascending aorta, which is a known derivative of cardiac neural crest cells [34]. Consistent with this observation is the significantly increased frequency of abnormal aortic valves in first-degree family members of patients known to have hypoplastic left heart syndrome. Given the role of neural crest cells in the development of the aortic valve and arch, these data suggest a potential role for impaired neural crest cell function in causing the syndrome. 

## 8. Coronary Arterial Hypoplasia and Proliferation of Cardiomyocytes

Patients with the syndrome in the setting of mitral stenosis, despite having thickening of the left ventricular walls, typically have a relatively diminutive left coronary arterial system. This contrasts with the dilated and enlarged left coronary arterial system observed in patients with hypertrophic cardiomyopathy. The latter finding is likely an adaptive response required to maintain an adequate arterial supply to the massively thickened myocardium. The basis for the relative coronary arterial hypoplasia in the setting of the mural thickening seen with hypoplastic left heart, however, remains unknown. Genetically engineered animal models for genes associated with the syndrome, nonetheless, show an abundance of left ventricular mural cardiomyocytes. With this setting during fetal life, the diminutive left coronary arterial system may well cause a relative mismatch between supply and demand, thus leading to myocardial ischemia and hypoxia. Numerous studies have demonstrated that hypoxia is a stimulus for cardiomyocytic hyperplasia [35]. Hence, the diminutive left coronary arterial system, leading to the supply–demand mismatch in the developing ventricular walls, may further exacerbate the cardiomyocytic hyperplasia.

## 9. Conclusion: Emerging Mechanistic Concepts in Hypoplastic Left Heart Syndrome

Findings in the genetically engineered animal models for *ETS1*, *Hand1*, *NKX2.5*, and *NOTCH1*, four genes known to be associated with the syndrome as found in humans, indicate an emerging common theme. This is the presence of hyperplasia of the cardiomyocytes during early cardiac development due to a disruption of the normal balance between the proliferation of the compact and trabecular layers of the ventricular walls. In the *ETS1* and *NOTCH1* mutants, furthermore, there was also the loss of an adjacent population of cells, specifically the pericardial cells in *Drosophila* and the endocardial cells in *Xenopus*. This suggests that these two genes function to establish a balance in the determination of the fates of the two cell types. Previous studies have demonstrated that another ETS family member, Etsrp/Etv2, is required for balancing the determination of the fates of the endocardial cells as opposed to the cardiomyocytes. The loss of Etsrp/Etv2 is known to cause a loss of endocardial cells and a gain of cardiac myocytes [36]. 

Together, these studies suggest a mechanistic link between the loss of endocardial cells, and hence reduced endocardial function, and cardiomyocytic hyperplasia. This could be achieved either by a direct effect on the determination of the fates of the cellular populations or by a perturbation of endocardial-to-myocardial signaling. 

Alternatively, the link could reflect hemodynamic changes arising from a primary defect in the development of either the aortic or mitral valves. Impaired flow into the developing left ventricle in consequence of an abnormality of the mitral valve could itself result in endocardial injury and dysfunction, thereby reconciling the flow theory for explanation of the syndrome. Another explanation could be that mutations in genes affecting the proliferation of the cardiomyocytes might themselves reflect a cardiomyocyte autonomous mechanism. This would involve cardiomyocyte-specific downstream pathways regulated by endocardial signaling. This “two-hit” model involving valvar and ventricular development would lead to a vicious cycle in which valvar development leads to impaired ventricular development and impaired ventricular development exacerbates impaired valvar development, or the other way around. The common endpoint could then be the phenotypic variant of the syndrome as seen in the setting of mitral stenosis. In either situation, there must be a factor, or factors, that predispose the ventricle to develop abnormally in the presence of endocardial dysfunction. Understanding the basis for why, in some cases, ventricular development during fetal life can proceed normally in the presence of aortic stenosis could provide critical insights into how the syndrome itself could be prevented. While the original results for therapeutic fetal balloon angioplasty have been disappointing [9], in retrospect, they are not surprising and are potentially informative. They likely reflect what would be predicted based on the animal models described above.

Future studies, therefore, should address the cellular composition of the ventricular walls in specimens showing the phenotypic features of the syndrome. We need to know how frequently the thickening of the walls is the consequence of cardiomyocytic hyperplasia as opposed to increased numbers of other cell types. If it is proven that hyperplasia is involved, we need to know whether is disruption of the balance between proliferation of the compact and trabecular zones. An important unanswered question is how, at least in some cases, a dilated thin-walled and poorly functioning left ventricle, as seen early in fetal development, evolves into the typical thickened left ventricle as seen in the variant of the syndrome with mitral stenosis. Another important issue is the relationship between the development of the valves and the left ventricle. The basis for the known variability in the human phenotypes is currently unknown. It is a critical area for future investigation if we are to fully understand the mechanistic relationships between impaired valvar and ventricular development.

Ultimately, it will be essential to identify the regulatory genetic networks affected in patients with the syndrome. Furthermore, the roles of the cardiac neural crest and the coronary arterial system remain to be explored. Complementing these studies will be the need to clarify the known complex genetics. To date, a disease-causing genetic mutation is known in only a very small subset of cases. It might well be thought that the recent investigation by Liu and colleagues [37] may have cast more light on this potential association. None of the mice mutants reported by these authors, however, possessed the phenotypic features of hypoplastic left heart syndrome as encountered in the human setting (see our Figure 1). Although the hearts exhibited aortic atresia and a hypertrophied left ventricle, the hearts did not show the features of hypoplastic left heart syndrome since none of the models showed the integrity of the ventricular septum in the setting of the concordant atrioventricular and ventriculo-arterial connections. Analysis of the images provided in Figure 1 of the report of Liu et al. [37,38,39] shows that the hypoplastic aorta arises from the roof of the right ventricle. The left ventricle in the specimen shown in Figure 1 [37], furthermore, shows none of the fibroelastotic changes that are anticipated in the human variant with mitral stenosis and aortic atresia (See again our Figure 1). It remains the case, therefore, that currently there are no mouse models that replicate the “classical” variants of hypoplastic left heart syndrome as encountered in human patients. This may be because the evidence accruing from analysis of the human variants, along with the findings from fetal echocardiography already discussed, supports the inference that the entity is an acquired disease of fetal life. Specifically, the morphological changes seen in the diseased hearts with mitral stenosis occur subsequent to commitment of the aortic root to the developing left ventricle and closure of the embryonic interventricular communication. It is, of course, potentially possible to follow such changes in a mouse model, but as emphasised, the changes would need to be shown to have occurred subsequent to closure of the embryonic interventricular communication. We are not ignoring the significant evidence existing to support the notion that there is a genetic component to the disease. It is premature, however, to presume that studies such as those reported by Liu and colleagues [37] have clarified the association. 

Thus, the current efforts of the Pediatric Cardiac Genomics Consortium [17], have yielded to date only a very small number of likely disease-causing genes for the syndrome. Future studies should help determine whether the remainder of the cases, which are currently considered to be idiopathic, are due to perturbations in additional genes in the known pathways. If this proves not to be the case, they will surely lead to the identification of novel pathways and hence provide the basis for new areas of investigation. Other efforts, such as by the Todd and Karen Wanek Family Program for Hypoplastic Left Heart Syndrome, may lead to new therapeutic approaches for the syndrome [40,41]. Much remains to be learned, therefore, about the pathogenesis of hypoplastic left heart syndrome. The studies described in this review reflect the complex, multifactorial etiologies including genetic, epigenetic, and environmental that likely must align to yield the different phenotypes that represent hypoplastic left heart syndrome as seen in the clinical setting. They also emphasize the challenges that remain in developing animal systems that accurately model the disease. Although for the majority of cases, the specifics are still unknown, there is cause for hope for understanding better the pathogenesis of this devastating disease.
